# University of Louisville

**Published:** 1846-08

**Authors:** 


### UNIVERSITY OF LOUISVILLE.

From an advertisement on the cover of our Journal, it will be seen that the Medical Institute of Louisville comes before the public, this season, under a new and more imposing name. It is now the Medical Department of the University of Louisville. The change of name does not, in any way, affect its organization; but it is the same school in Faculty, buildings, and all the apparatus and means of instruction. Under its new name the school enters in November upon its tenth year. It is now able to refer to a numerous host of graduates as the fruits of its labors, and is willing to be tried by the scriptural rule: "Give her of the fruit of her hands, and let her own works praise her in the gates."

Other departments, it is expected, will be organized and put in operation, ere long, when we shall have in reality, as we now have on paper, a university in Louisville. Meantime the medical school has gained by the new charter. Before, it was subject to agitatation, and every year brought attempts at change; now it rests upon a basis of security, and has only to go forward in the cultivation and advancement of medical science and learning. Its career, thus far, has been prosperous, and with its faculty will be the fault if its prosperity be less in coming years.

